# Excess mortality in northern Haiti during the 2010 cholera epidemic

**DOI:** 10.1371/journal.pntd.0011750

**Published:** 2023-12-06

**Authors:** Macceau Medozile, Gina S. Lovasi, Sergios-Orestis Kolokotronis, Lori A. Hoepner

**Affiliations:** 1 Department of Environmental and Occupational Health Sciences, School of Public Health, SUNY Downstate Health Sciences University, Brooklyn, New York, United States of America; 2 Urban Health Collaborative, Dornsife School of Public Health, Drexel University, Philadelphia, Pennsylvania, United States of America; 3 Department of Epidemiology and Biostatistics, School of Public Health, SUNY Downstate Health Sciences University, Brooklyn, New York, United States of America; 4 Institute for Genomics in Health, SUNY Downstate Health Sciences University, Brooklyn, New York, United States of America; 5 Division of Infectious Diseases, Department of Medicine, College of Medicine, SUNY Downstate Health Sciences University, Brooklyn, New York, United States of America; Wayne State University, UNITED STATES

## Abstract

In the course of infectious disease outbreaks, barriers to accessing health care can contribute to preventable mortality. According to the Ministry of Health of Haiti (Ministère de la Santé Publique et de la Population [MSPP]), the 2010 cholera epidemic caused 7,936 deaths from October 2010 to December 2012 in Haiti alone. We seek to quantify the excess mortality attributable to patients not seeking care during the cholera outbreak in the Nord Department in 2010–2012. Using data from a community-based retrospective survey conducted by Doctors Without Borders (Médecins Sans Frontières [MSF]) in Northern Haiti, we used logistic regression to examine the association between healthcare utilization and fatality among household members with watery diarrhea in the Communes of Borgne, Pilate, Plaisance, and Port-Margot in the Nord Department. We found that failing to seek care resulted in a 5-fold increase in the case fatality ratio among infected individuals (26%) versus those who sought care (5%). Common concerns noted for why care was not sought included travel distance to treatment centers, not attributing watery diarrhea episodes to cholera, and being unsure where to seek health care for their watery diarrhea episodes within their Communes. In conclusion, addressing transportation and information needs could increase healthcare utilization and reduce lives lost during an outbreak.

## Author summary

The cholera epidemic in Haiti was unprecedented. One month after the first case was confirmed, the Haitian government quickly activated a national cholera surveillance system (NCSS) to gather relevant epidemiologic information on the disease to inform interventions. Although oral cholera vaccine (OCV) was not available in all administrative departments during the first two years of the epidemic, care seeking for watery diarrhea was increasing throughout the country. However, certain deficiencies relating to determinants of health have prevented many from seeking care in hospitals and clinics, or in numerous cholera treatment centers (CTC) and oral rehydration points (ORP) established in every administrative department in the country. To estimate the effects of those determinants of health and health behaviors on cholera fatality in the Nord Department, we used data from a retrospective survey conducted in the Communes of Borgne, Pilate, Plaisance and Port-Margot by Doctors Without Borders (Médecins sans Frontières, MSF) in 2011. We found that failing to seek care was associated with death, and that common reasons for not seeking care included concerns about travel distance to CTCs and uncertainty about where to seek health care in those Communes for watery diarrhea symptoms. Establishing CTCs and ORPs within rural communities located in mountainous areas with lack of road accessibility could have reduced the excess mortality attributable to not seeking healthcare. In addition, although the Haitian government and the World Health Organization (WHO) declared the country free of cholera in 2020, risk factors associated with the rapid spread of the infection in 2010 have ongoing relevance to population health and vulnerability to future outbreaks.

## 1. Introduction

In the context of an ongoing disease outbreak, barriers to care-seeking can contribute to preventable fatality. Impediments such as lack of trust in the public health system, misperceptions [1] and poor public health infrastructure [2] have delayed care-seeking for Ebola and caused many preventable deaths during the 2014–2016 outbreak in Sierra Leone. Similarly, one study on cholera outbreak in West Kenya suggested that cholera patients with bloody diarrhea who sought care at health centers operated by their government had lower odds of survival when compared to those who self-treated at home [3]. In addition, in Karachi, Pakistan, a study found higher odds of fatality among children with bloody diarrhea for those whose parents refused their children care versus allowed their children to be admitted to a hospital [4].

In 2020, the Haitian government and the World Health Organization (WHO/PAHO) marked the end of the cholera epidemic, which caused 9,792 fatalities in Haiti [5]. The outbreak had first been confirmed by the country’s ministry of public health (MSPP) and the Centers for Disease Control and Prevention (CDC) in October 2010, with confirmed infections and hospitalizations throughout the 10 administrative departments of the country a month later. Cholera initially spread from the Artibonite Department, where the Artibonite River was infected with fecal matter embedded with *Vibrio cholerae* by United Nations Peacekeeping soldiers [6,7,8,9,10,11]. Vulnerability to the outbreak was increased by poor public health infrastructure and an inadequate number of physicians and nurses to meet the demand during the crisis, a concern already identified in 1998, when only about 1 physician was available per 4,000 people [12]. Lack of public health services in rural areas and a poor water and sanitation system facilitated a rapid spread of the infection throughout the country.

The Haitian Ministry of Health (MSPP) created a national cholera surveillance system (NCSS) in November 2010 to collect and report cholera case counts and fatality rates. NCSS provides data to map out the spread of the infection and inform governmental strategy to provide health services to cholera victims through cholera treatment centers (CTC) established within hospitals and health centers. NCSS compiled daily reported data from cholera treatment facilities, community health workers, local physicians, and information provided through word-of-mouth from local leaders [13]. During the last century, Haiti was the only country where a cholera epidemic has caused such high rates of infection in a short period of time. Worldwide, in 2019, cholera represented an estimated burden of 1.4 to 4.0 million cases, and 21,000 to 143,000 deaths per year [14]. The Haitian epidemic has caused higher case fatality rates than the 1991–2009 Peruvian and Bolivian cholera epidemics combined [15,16]. In fact, the cholera epidemic in Haiti accounted for 57% of all cholera cases and 53% of all cholera deaths worldwide reported to the WHO during 2011 [13].

The rapid intervention by MSPP through NCSS allowed officials to assess the severity of the infection and develop series of interventions to control its impacts. Nevertheless, NCSS data may have underestimated cases due to internal migration for education, commerce and health services, and stigmatization may have contributed to incomplete ascertainment of cases. A study [17] reported that 87% of the cholera-specific fatality occurred outside of the established health centers which were required to report cases, even though over 50% of all cholera patients nationwide were estimated to have received treatment in MSF-operated facilities.

Despite the rapid intervention of Haiti’s health department, environmental and other socio-economic conditions have impacted the national implementation of water, sanitation and hygiene (WASH) improvements and related mass education programs [18]. Moreover, the country’s poor healthcare infrastructure, the absence of health services and latrines in many communities, and poor road conditions were obstacles to effective and timely control of the epidemic. This study sought to quantify the relative contribution of preventable mortality due to not seeking care among those experiencing watery diarrhea symptoms during the 2010–2012 cholera outbreak in the Nord Department of Haiti.

## 2. Methods

### 2.1 Ethics statement

Doctors Without Borders (MSF) were granted approval by the Haitian Ethics Committee and MSPP to conduct the survey in the Nord Department. The institution followed the ethical principles recommended by the Declaration of Helsinki. Written informed consent was obtained from all participants of the study [19].

### 2.2 Study design

According to the Institut Haitien de Statistiques et d’Information (Haitian Statistics Institute), the country is divided into 10 administrative departments, 42 Arrondissements, 140 Communes and 570 Communal Sections. The latter is the smallest administrative division of Haiti. A village is located within a Communal Section. We used publicly available data (S1 Data) from a two-stage, household-based cluster survey conducted by Doctors Without Borders (MSF) in 138 clusters of 23 households in four communes of the Nord Department, Haiti from April 22^nd^ to May 13^th^, 2011 [19] (the recall period was 170 days). The primary data were collected using a standardized questionnaire, administered in Creole to heads of households (mean household size: 5 people) present during the assessment period. Respondents provided information on sex, age, severity of watery diarrhea episodes, and cholera fatality among household members who were present at the beginning of the recall period. Heads of household were excluded from the fatality analysis. During that survey, 3,187 households in 138 clusters were randomly visited. The sample size for this study is 16,000 people. Based upon available data from the United Nations World Population Prospects [20], the estimated crude mortality rate for Haiti in the absence of an epidemic in 2010 was 17.738/1,000 (person-year) and 8.762/1,000 (person-year) in 2011 during the epidemic.

### 2.3 Survey measures

**Fig 1:**
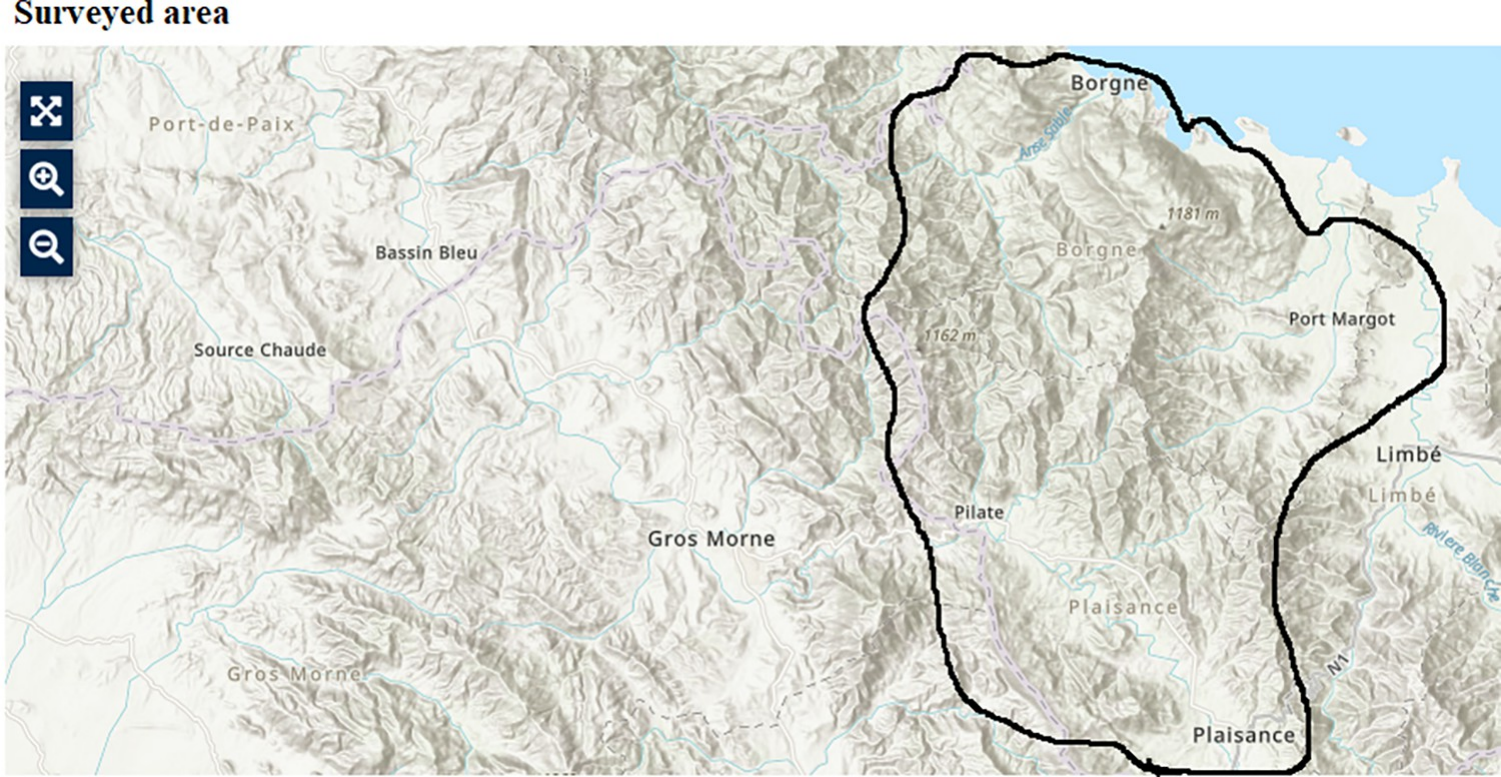
Map of Haiti: Surveyed Population– 2010–2011.

The head of the household was asked to report the reasons for which relevant household members did not seek health care. Villages with more than 5,000 inhabitants were considered urban and excluded from the survey [19]. The surveyed population was a representative sample of the Nord Department. The four communes surveyed included Borgne, Pilate, Plaisance, and Port-Margot, spanning 3 out of 7 Arrondissements of the Nord Department (Fig 1). These selected communes were similar to the entirety of the Nord Department: approximately 50% of the population was under the age of 18, and gender distribution was similar to that of the department as a whole. Two of the communes had access to roads (N = 108,638) and the others were more difficult to reach (N = 110,011) because of the steepness of the terrain (Borgne and Pilate) [21].

### 2.4 Specific variables

For this study, reasons for not seeking-health care were coded as follows: 1) concerns about cost (yes/no), 2) unsure where to seek care (yes/no), 3) concerns about travel distance (yes/no), 4) did not attribute symptoms to cholera (yes/no). For those who sought care, providers were categorized as 1) cholera treatment centers (CTC) operated by MSF, 2) non-MSF-CTC (not operated by MSF), 3) hospitals or clinics, and 4) oral rehydration points (ORP). In addition, means of transportation to treatment centers were coded as 1) by motorized vehicle or 2) by foot; the latter included instances of being carried on another person’s shoulders.

### 2.5 Statistical analysis

Data from the cross-sectional survey were analyzed using SPSS version 21. We used logistic regression analysis (adjusted for age and sex) to examine the association between seeking care and fatality among those household members with watery diarrhea and when reported as the outcome of the most severe diarrheal episode based upon MSF’s initial criteria [19]. Excess deaths were calculated as the difference between observed deaths among cases who did not seek health care and the estimated crude death rate for 2011 (during the epidemic) and 2010 (before the epidemic) for Haiti by the United Nations World Population Prospects [20] In addition, we described commonly reported reasons for not seeking care to measure attributable fatality due to cholera.

## 3. Results

### 3.1 General description

The Communes of Borgne and Plaisance represent 66.0% (N = 9,613) of the total surveyed population. The mean age of the household members within the four surveyed communes was 27 years old, and women constituted 52.3% (N = 8,844) of that population. The number of reported diarrhea fatalities in the communes within a year of the first national cholera case was 224 people. Among those with at least one watery diarrhea episode (n = 1977), 88.8% survived, and the mortality rate differed geographically: 64.2/1000 deaths were reported in the commune of Borgne, 28.5/1000 in Pilate, 10.8/1000 in Plaisance and 6.9/1000 in Port-Margot. About 43% (N = 850) of these cases occurred in the Commune of Borgne, with a mean age of 31 years old among males and 30 years old for females.

We found that watery diarrhea CFR was higher among females than their male counterparts in Borgne (male = 14.7% and female = 15.9%) and Port-Margot (male = 1.51% and female = 8.95%). Of those cases who did not seek care for their diarrhea episodes, CFRs among female were lower than their male counterparts in Pilate (male = 29.8%, female = 21.0%) and Plaisance (male 12.7%, female = 8.33%). CFR among cases 60 years of age or older were about three times higher than those in the group age of 20–29 and 1.5-times greater than those in the group age of 30–39. In addition, CFR among cases who did not seek care was five times higher (26.0%) than their counterparts who sought care (5.0%). The death ratio from watery diarrhea was higher among those who traveled by foot (73.2/1000) than their counterparts (37.6/1000).

### 3.2 Mortality among patients who sought care

The percent fatality among patients who sought care at the four categories of healthcare providers was estimated at 5.12% (N = 1,426), those who were 60+ years old (N = 347) were more affected (37%). Patients who received care at ORPs (N = 44) were shown to have lesser risks (CFR = 4.5%) of dying from their watery diarrhea episodes versus those who did not seek care (Table 1). Also, higher CFR was noted among cases who sought care at clinics not operated by MSF (6%) than their counterparts. Overall, within the surveyed population, the percent fatality among patients who sought care in the communes of Borgne and Pilate was 79.3% (N = 188).

**Table 1:** Demographic Distribution of all Reported Cases of Watery Diarrheal Episodes and Fatality in the Communes of Borgne, Pilate, Plaisance and Port-Margot: Haiti 2010–2012 (N = 2,032).

		Diarrheal Cases		Deaths		CFR%
		N	(%)	N	(%)	
Sex						
	Male	969	(48.4	105	(46.9)	10.8
	Female	1035	(51.6	119	(53.1)	11.2
Age						
	<5	272	(13.4)	18	(8.0)	6.6
	6 to 9	172	(8.5)	14	(6.3)	8.1
	10 to 19	428	(21.0)	26	(11.6)	6.1
	20–29	258	(12.7)	21	(9.4)	8.1
	30–39	202	(9.9)	27	(12.1)	13.4
	40–49	183	(9.0)	19	(8.5)	10.4
	50–59	172	(8.5)	17	(7.6)	9.9
	60 plus	347	(17.1)	82	(36.6)	23.6
Communes						
	Borgne	850	(41.8)	130	(58.0)	15.3
	Pilate	490	(24.1)	58	(25.9)	11.8
	Plaisance	423	(20.8)	22	(9.8)	5.2
	Port-Margot	271	(13.3)	14	(6.3)	5.2
Health Seeking Behaviors						
	Did not seek care	583	(28.7)	151	(67.4)	26
	Sought care	1447	(71.3)	73	(32.6)	5
Health Providers						
	MSF—CTC	773	(54.2)	41	(56.2)	5.3
	Non-MSF CTC	101	(7.1)	6	(8.2)	6
	Hospital or Clinics	508	(35.6)	24	(32.9)	4.7
	ORP	44	(3.1)	2	(2.7)	4.5
Transport Means						
	Motorized Vehicles	1189	(58.8)	76	(33.9)	6.4
	By Foot	832	(41.2)	148	(66.1)	17.8

MSF-CTC = Cholera treatment centers operated by Doctors Without Borders

Non-MSF-CTC = Cholera treatment centers not operated by Doctors Without Borders

ORP = oral hydration points

### 3.3 Reported mortality and health behaviors

**Fig 2:**
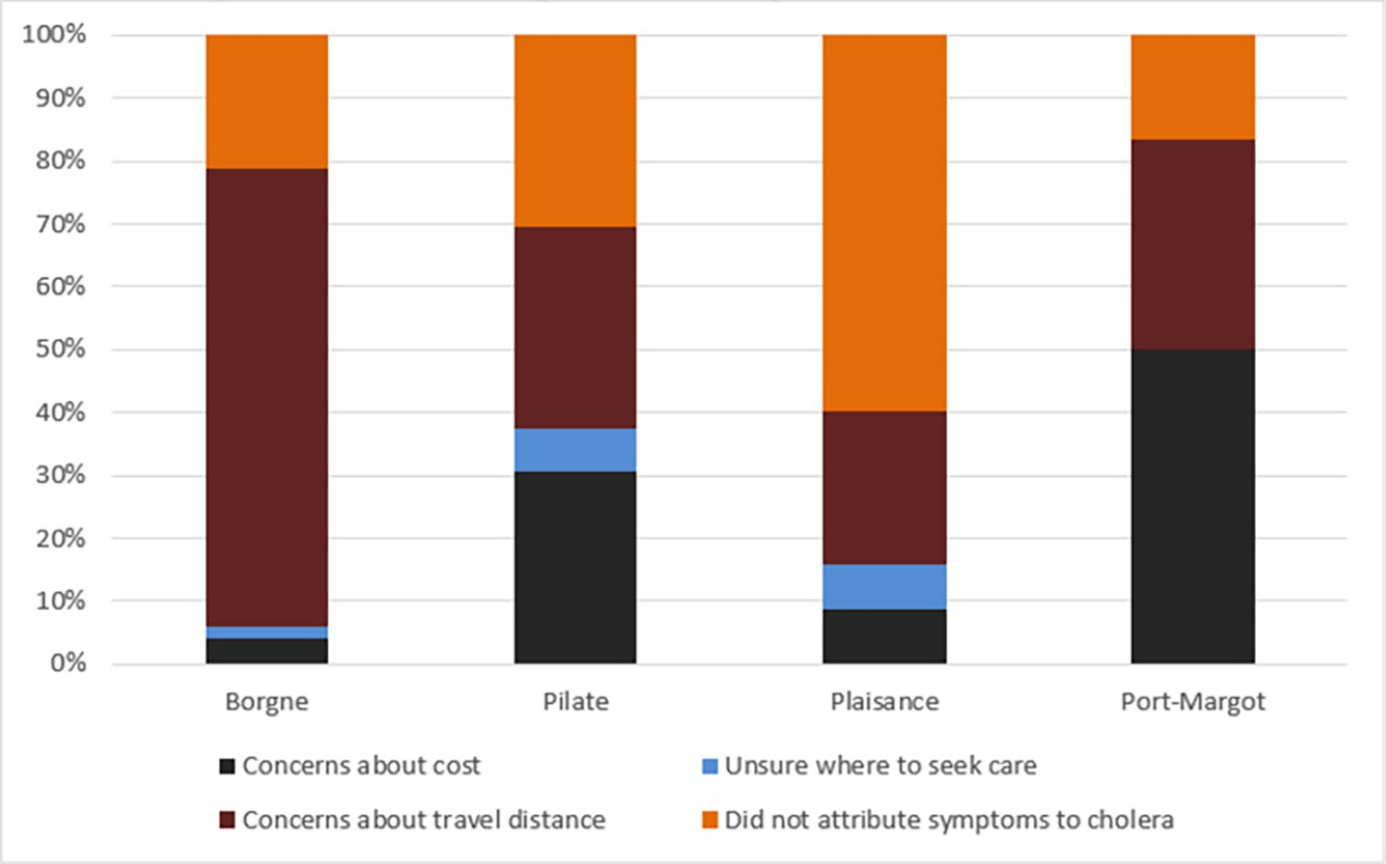
Commonly Reported Reasons for Not Seeking Health Care in the Nord Department Haiti during the Cholera Epidemic in 2010.

Concerns about travel distance to CTCs and not attributing diarrhea symptoms to cholera were found to be the most common reasons for not seeking care in the Communes of Borgne, Pilate and Plaisance (Fig 2). Similarly, concerns about health care costs held cholera victims back from seeking care in Pilate and Port-Margot.

Higher percentages of watery diarrhea-specific fatalities (Table 2) were significantly associated with cases with concerns about distance travel (OR = 8.90, CI: 7.21, 11.23) to CTCs in the four surveyed communes. In addition, not attributing watery diarrhea episodes to cholera (OR = 1.79, CI: 1.17, 2.74) increased the odds of fatality among cases who did not seek health care in the Nord Department.

**Table 2:** Watery Diarrhea-Specific Mortality among cases Not Seeking-Health Care in the Nord Department: Haiti 2010–2012 (N = 143).

Reasons for not seeking care	Fatality n (%) *p-value* OR 95% C.I.				
Concerns about cost	5 (3.5)	0.49	0.96	0.41	2.23
Unsure where to seek care	5 (3.5)	0.49	0.98	0.42	2.27
Concerned about travel distance	114 (80.0)	0.01	8.90	7.21	11.23
Did not attribute symptoms to cholera	19 (13.0)	0.01	1.79	1.17	2.74

### 3.4 Excess mortality in the surveyed communes

There were 143 fatalities in a population of 373 cases, representing the four communes surveyed in the Nord Department where respondents reported specific reasons and concerns for not seeking healthcare (Table 3) for their watery diarrhea episodes. Based on the United Nations World Population Prospects, in 2011, the expected number of fatalities among those 373 cases who did not seek care would be 3.27 per 1,000. However, the excess deaths attributed to failure to seek health care was about 136/1,000 people in 2010 and 140/1,000 in 2011. Concerns about health care costs and uncertainty as to where to seek care caused 9 preventable deaths/1,000 in the 4 Communes. In addition, there was an excess of approximately 112/1,000 deaths among cases who were concerned about travel distance to CTCs in the surveyed population.

**Table 3:** Unadjusted Relative Contribution of Preventable Mortality Among Cases Experiencing Watery Diarrhea Who Did Not Seek Care in the Nord Department, Haiti: 2010–2011 (N = 373).

Standardized Mortality Ratio							
		Observed deaths "(O)"	Expected deaths* "(E2010)"	Expected deaths** "(E2011)"	SMR "(O/E)"	Excess deaths "(O—E2010)"	Excess deaths "(O—E2011)"
**Reasons for not seeking care.**	Population						
All reasons and concerns	373	143	7	3	21.61	136	140
Concerns about cost	47	5	1	0	6	4	5
Unsure where to seek care	13	5	0	0	21.68	5	5
Concerns about travel distance	213	114	4	2	30.17	110	112
Did not attribute symptoms to cholera	100	19	2	1	10.71	17	18

Expected crude death rate for 2010* (17.738/1000) and 2011**(8.762/1000) for Haiti by the United Nations World Population Prospects

## 4 Discussion

In general, of the communes surveyed in the Nord Department, failing to seek healthcare resulted in higher case fatality ratio values for watery diarrhea. Thus, when compared with patients who sought care for their watery diarrhea episodes, the odds of survival were significantly lower among cases who did not seek health care.

**Fig 3:**
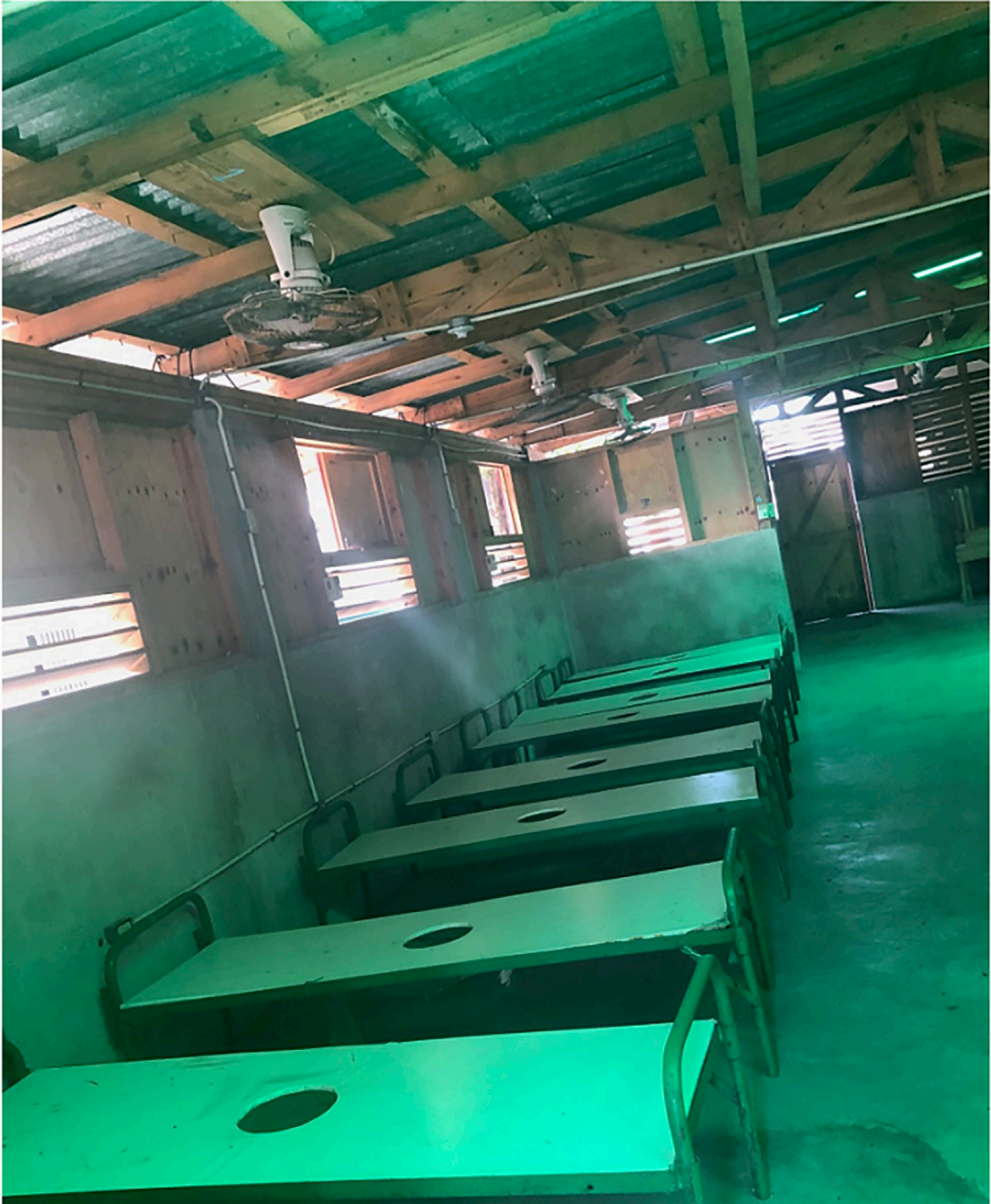
Cholera treatment center at Les-Cayes General Hospital, Sud Department in July 2019.

Concerns about travel distance (by foot) to CTCs were among the chief factors for not seeking healthcare throughout the communes. In addition to difficult terrain (non-drivable mountainous roads) to reach CTCs from the villages, during the hurricane season, mudslides and floods make it even more difficult to cross the rivers and carry a sick person on men’s shoulders for many kilometers. Those trips were very dangerous and could require more than a day of walking. This explains why CFRs for watery diarrhea in the Communes of Borgne (135 meters above average elevation of Haiti) and Pilate (131 meters above the average elevation of Haiti) were so elevated and the differences attenuated with adjustment for care-seeking. The average elevation of Haiti is 470 meters above sea level (Haiti: country data and statistics, April 5, 2023). Had the CTCs been located within or closer to the case clusters, the excess death could have been reduced in the communes (Fig 3). Some of the CTCs were operated in mobile tents and could have been relocated to the steepest geographic locations in Borgne and Pilate.

In addition, lack of messaging to the more remote villagers in the communes seemed to be an important factor for not seeking health care. Our study found that among those who were unsure where to seek care for their watery diarrhea episodes, excess fatality could have been prevented had a more effective participatory public education campaign been developed and implemented in the communes. Along with other influential community members, women organizations and religious leaders in the far-reaching rural populations should be trained to disseminate effective public health messages throughout their communities. Moreover, Haitian’s “*marché publique*” (public market) in rural settings is a powerful social platform to launch health education campaigns and recruit volunteers to help improve community health [22,23,24,25]. This could also treat any distrust of health care practitioners and facilities. The use of remote sensing technology could have helped Haitian decision-makers to provide faster and more direct healthcare to those living in rural areas during the early stage of the epidemic. A study conducted in Haiti [26] showed how mobile phone data were used to analyze mobility patterns, and to identify and even anticipate local outbreaks of cholera at the beginning of the epidemic in Haiti.

Our results contribute to a growing body of evidence that provides the basis for effective strategies to establish adequate and equitable distribution of healthcare services in Haiti and other developing countries. In Haiti, recent political, socio-economic, and environmental issues, such as rampant armed gang activity, kidnappings of civilians and healthcare providers, which led to the closing of health care centers and hospitals, along with massive community displacement, unsafe public transportation, and basic sanitation issues make the country more vulnerable to the resurgence of cholera and other communicable diseases.

### Limitations

Recall bias may have limited the accuracy of data used in our analysis, since the retrospective survey conducted by MSF in the communes took place six months after the first cholera case was identified. Because of the stigmatization of the infection and the pain that it caused to talk about loss of loved ones barriers to care seeking, the number of fatalities may have been under-reported. In addition, the clusters may have not accurately represented the distribution of the infection and the associated fatalities observed and reported by the locals in the early days of the epidemic because of internal migration relative to school attendance/education, employment opportunity, natural disasters, political and human rights violations within neighborhoods and communities throughout the country.

### Conclusions

Our study found that people who lived in the far-reaching communes and those who did not seek care because they did not attribute their watery diarrhea episodes to cholera were at higher risk for death in the Nord Department. Those factors also contributed to excess fatality in the surveyed communes. Women and older adults carried a higher burden than the rest of the population. We recommend more strategic and continuous training for healthcare field personnel and private doctors to better handle the resurgence of the infection in Haiti. It is important to demystify the infection and develop direct and applicable strategies with women’s organizations and local religious leaders, among others, to promote public health education, particularly in remote communities. It is equally important that the health facilities damaged during the earthquakes of 2010 and 2021 be repaired and operational to improve public health conditions in Haiti in general.

## Supporting information

S1 Data Supplementary dataset. (XLSX)
